# Peptide–siRNA Supramolecular Particles for Neural Cell Transfection

**DOI:** 10.1002/advs.201801458

**Published:** 2018-12-05

**Authors:** Armando Hernandez‐Garcia, Zaida Álvarez, Dina Simkin, Ashwin Madhan, Eloise Pariset, Faifan Tantakitti, Oscar de J. Vargas‐Dorantes, Sungsoo S. Lee, Evangelos Kiskinis, Samuel I. Stupp

**Affiliations:** ^1^ Simpson Querrey Institute Northwestern University Chicago IL 60611 USA; ^2^ Department of Chemistry of Biomacromolecules Institute of Chemistry National Autonomous University of Mexico Ciudad Universitaria Mexico City 04510 Mexico; ^3^ Department of Pharmacology Feinberg School of Medicine Northwestern University Chicago IL 60611 USA; ^4^ The Ken & Ruth Davee Department of Neurology & Clinical Neurological Sciences Department of Physiology Feinberg School of Medicine Northwestern University Chicago IL 60611 USA; ^5^ Department of Chemistry Northwestern University Evanston IL 60208 USA; ^6^ Department of Materials Science and Engineering Northwestern University Evanston IL 60208 USA; ^7^ Department of Biomedical Engineering Northwestern University Evanston IL 60208 USA; ^8^ Department of Medicine Northwestern University Chicago IL 60611 USA

**Keywords:** glial fibrillary acidic protein (GFAP), glial cells, knockdown, neurons, protein engineering, supramolecular particles, synaptophysin, transfection

## Abstract

Small interfering ribonucleic acid (siRNA)‐based gene knockdown is an effective tool for gene screening and therapeutics. However, the use of nonviral methods has remained an enormous challenge in neural cells. A strategy is reported to design artificial noncationic modular peptides with amplified affinity for siRNA via supramolecular assembly that shows efficient protein knockdown in neural cells. By solid phase synthesis, a sequence that binds specifically double‐stranded ribonucleic acid (dsRNA) with a self‐assembling peptide for particle formation is integrated. These supramolecular particles can be further functionalized with bioactive sequences without affecting their biophysical properties. The peptide carrier is found to silence efficiently up to 83% of protein expression in primary astroglial and neuronal cell cultures without cytotoxicity. In the case of neurons, a reduction in electrical activity is observed once the presynaptic protein synaptophysin is downregulated by the siRNA–peptide particles. The results demonstrate that the supramolecular particles offer an siRNA delivery platform for efficient nonviral gene screening and discovery of novel therapies for neural cells.

The development of efficient, easy‐to‐use and nontoxic molecular carriers for delivery of small interfering ribonucleic acid (siRNA) into neural cells (neurons and glial cells) would accelerate the functional screening of genes and consequent discovery of therapeutic targets for neurodegenerative diseases.^1^, ^2^, ^3^, ^4^ Transfection of primary neuronal cells, however, is challenging in part due to low survival rate in vitro and their lower nanoparticle uptake compared to neuronal cell lines.^5^ At the same time, there is a lack of appropriate nonviral methods that integrate high transfection efficiency, low cytotoxicity, biodegradability, and scalability.^6^, ^7^ Peptides have been of interest as siRNA delivery systems since they are structurally well defined, biodegradable, often nontoxic, and can be prepared through cost‐effective synthetic methods.^8^ With peptides there is also the possibility to incorporate bioinspired sequences that specifically recognize nucleic acids or proteins for targeting purposes.^8^, ^9^, ^10^, ^11^, ^12^, ^13^ One of the common strategies has been the use of cell‐penetrating peptides as carriers of siRNA; however, these molecules have nonspecific electrostatic interactions with siRNA and tend to be toxic as a result of their high positive charge.^14^

In this work, we report on a supramolecular particle that effectively transfects neural cells with very high efficiency and with minimal toxicity. We utilized the nonelectrostatic and specific RNA recognition ability of a peptide taken from a double‐stranded RNA‐binding domain (dsRBD) in *Xenopus laevis* conjugated to a self‐assembling domain to generate sub‐micrometer supramolecular particles in aqueous solution with siRNA. These supramolecular particles transfect and knockdown efficiently protein expression in primary neural cultures without significant toxicity. The easily prepared peptide particles can be internalized by primary neural cells and silence efficiently protein expression. We also tested the platform by evaluating the reduction of expression of a synaptogenic protein using electrophysiology experiments.

We utilized for our strategy a segment of 28 amino acids (referred to as P2) present in a dsRBD of proteins in the African clawed frog *X. laevis*.^15^ Peptide P2 is one out of three siRNA‐binding regions present in the full dsRBD. This particular region with the sequence TSKQVAK is known to interact across the major groove of the dsRNA.^15^ We chose not to include the other two regions since they are contained within a very hydrophobic sequence (referred to as P1) that was found to have poor water solubility (**Figure** **1**a).

**Figure 1:**
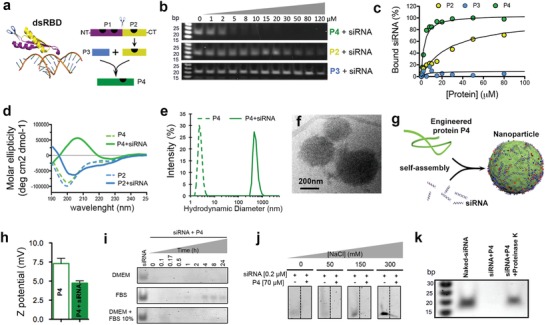
Design of peptide with amplified siRNA binding via self‐assembly. a) Scheme showing crystal structure of a dsRNA‐binding domain from *X. laevis* (see ref. 15) (left). C‐terminus RNA binding motif P2 (in yellow) was extracted from the dsRBD and fused to a self‐assembly enhancer peptide P3 to obtain P4, a peptide with amplified siRNA binding (right). Black marks on the peptide schematics are dsRNA‐binding sites according to the crystal structure. b) Electrophoresis gels of peptides bound to siRNA. c) Plot of percentage of peptide‐bound siRNA derived from the electrophoresis gels. d) Plot of molar ellipticity (circular dichroism) of P4 and P2 peptides alone (dashed lines) and with siRNA (solid lines). e) Hydrodynamic size of P4 peptide alone (dashed line) and with siRNA (solid line). f) P4–siRNA particles in solution imaged by cryo‐TEM. g) Schematic representation of an unstructured P4 polypeptide that binds siRNA to assemble into particles. h) ζ‐potential of P4 peptide alone (empty bar) and with siRNA (solid bar). i) Electrophoresis gels showing the release and digestion of siRNA bound to peptide P4 when incubated in different media, DMEM (top), FBS 100% (middle), and DMEM + FBS 10% (bottom). j) Electrophoresis gels showing the release of siRNA from siRNA–P4 particles in aqueous solutions as a function of NaCl concentration. k) Electrophoresis gel showing release of siRNA from P4 particles when incubated with proteinase K. Molecular marker (first lane from left to right), naked siRNA (second lane), siRNA–P4 complexes (third lane), and siRNA–P4 complexes incubated with proteinase K for 24 h (fourth lane).

We fused P2 to a peptide segment called P3 (18 amino acids + a spacer of two glycines) to obtain the peptide sequence called P4 (Figure 1a; for full amino acid sequences and characterization, see Figure S1 and Table S1 in the Supporting Information). P3 is known to undergo structural switching from α‐helix to β‐sheet upon binding to Cu^2+^ ions leading to the formation of aggregates.^16^ The rationale for fusion was that siRNA binding to P2 and possibly to P3 could lead to a similar structural switch and promote aggregation of peptides and siRNA. We expected, of course, siRNA would bind to P2 but also to the four cationic residues in P3 (one K and three R residues). Using gel electrophoresis we found that P4 exhibited a tenfold increase in siRNA‐binding strength compared to P2, whereas P3 failed to bind siRNA altogether (Figure 1b,c). The lack of binding of siRNA to P3 could be due to its conformation and electrostatic repulsion from its anionic amino acids. However, a synergy for siRNA binding obviously occurs when P2 and P3 are fused to create P4. We also found that the binding strength of P4 to siRNA was comparable to that of dsRBD (Figure S2a–c, Supporting Information). These findings suggest that fusion of the peptide P2 to P3 helped to encapsulate siRNA, generating an effective binding capacity comparable to the much larger peptide dsRBD. Interestingly, P4 binds strongly to any double‐stranded nucleic acid but very poorly to single‐stranded nucleic acids (Figure S2d, Supporting Information). Hence, P4 can be used for encapsulation of dsRNA or dsDNA. Further characterization with circular dichroism revealed that P4 switches from a random coil to β‐sheet secondary structure upon interaction with siRNA (Figure 1d), a transition not observed in the case of P2. This observation supported our initial hypothesis that P3 could act as a structural switch possibly leading to peptide–siRNA aggregation. A peptide, referred to as P5, known to exhibit a similar conformational change^17^ as P3 also revealed similar behavior as P4 when interacting with siRNA after fusion to P2 (resultant peptide was P5*–*P2; see Figure S3, Supporting Information). Again, this observation supports the peptide design hypothesis mentioned before as the strategy to bind siRNA.

Using dynamic light scattering (DLS), small angle X‐ray scattering (SAXS), and cryo‐transmission electron microscopy (cryo‐TEM), we found that peptide P4 forms small aggregates (DLS: 3 nm, SAXS: 16 nm), and upon addition of siRNA, we observed the appearance of spherical supramolecular particles measuring 200–300 nm based on cryo‐TEM (Figure 1e,f; Figure S2e and Table S2, Supporting Information). We also confirmed this observation using super‐resolution structured illumination microscopy (SIM) and atomic force microscopy (AFM) (Figure S2f, Supporting Information). In the case of P2, we observed the presence of small particles (<20 nm) after the addition of siRNA (data not shown). We therefore attribute the formation of the peptide–siRNA supramolecular particles to the random coil to β‐sheet structural change that occurs upon contact with the nucleic acid (see Figure 1g). We hypothesize that this structural change exposes hydrophobic amino acids from P4 at the interface of the peptide–siRNA complex with water, thus triggering self‐assembly into nanoparticles through hydrophobic collapse. However, further studies beyond the scope of this manuscript are needed to unravel the complex interaction between the peptide and siRNA.

We followed changes in the circular dichroism spectrum of P4 upon addition of siRNA, and observed the gradual disappearance of the random coil peak at 200 nm and the simultaneous appearance of a negative peak at 220 nm and the positive peak at 195 nm characteristics of β‐sheets (Figure S2g,h, Supporting Information). The intensity of the β peaks reached a plateau value at a molar ratio of peptide‐to‐siRNA equal to 12, suggesting that one siRNA molecule is interacting on average with 12 P4 peptide molecules (Figure S2i, Supporting Information).

The ζ‐potential (zeta potential) of P4 remains low before and after forming particles with siRNA (+7.3 and +4.7 mV, respectively) (Figure 1h), which reflects the low charge density of P4, the presence of both cationic and anionic residues in P4, and the high molar ratio of peptide to siRNA in the supramolecular particle. This is beneficial since high ζ‐potential particles have been associated with cytotoxicity.^18^ However, the slightly positive potential can still contribute to the colloidal stability of the supramolecular particles. Next, we demonstrated that P4–siRNA particles are highly stable and resistant to enzymatic degradation when incubated with Dulbecco's modified Eagle's medium (DMEM), 100% fetal bovine serum (FBS), and DMEM + 10% FBS (Figure 1i). We also did not observe release of siRNA from the particles in solutions containing a high salt concentration (Figure 1j). Interestingly, lower salt and enzymatic stability were observed when P4 complexed dsDNA (Figure S2j,k, Supporting Information) suggesting that P4 is better suited for siRNA than short dsDNA. By DLS we did not observe the disassembly of the particles P4–siRNA in the presence of either buffer, DMEM, or DMEM+ 10% FBS (Figure S2l, Supporting Information). The stability of the P4–siRNA particles in high ionic strength solutions suggests that electrostatic interactions are not the only driving force for siRNA encapsulation. This was corroborated by the fact that we did not observe release of siRNA when particles were incubated in the presence of heparin sulfate at concentrations found in human plasma (Figure S2m, Supporting Information). Finally, we found that encapsulated siRNA by P4 can be released after protease digestion of the supramolecular particles, which suggests a possible release mechanism inside cells (Figure 1k).

To understand how these supramolecular particles function, we explored the use of the siRNA–P4 supramolecular particles to transfect primary cortical neural cells in vitro. For that purpose, postnatal astroglial cells and embryonic neurons were used since they are difficult to transfect, and the two types of cells make up the majority of the cells present in the central nervous system (CNS). We first quantified the transfection efficiency of a fluorescent siRNA–Alexa488 internalized by the peptide P4 particles using flow cytometry and confocal imaging. As nonviral siRNA positive delivery controls, we used the commercially available vehicles Lipofectamine (cationic lipids) and N‐TER (peptide) to transfect astrocytes (**Figure** **2**a) and neurons (Figure 2b). After 24 h of incubation, the siRNA–P4 (P4) supramolecular particles transfected glial (74% ± 3.3%) and neuronal (78.5% ± 4%) cell cultures seeded for 15 days in vitro. The total number of glial cells transfected was higher for P4 than for N‐TER (34.3% ± 6.4%) but slightly lower relative to Lipofectamine (89% ± 1.4%). However, based on the mean fluorescence intensity of the astroglial cells, P4 transfected more efficiently (2.1 = 10^4^ mean fluorescence) than Lipofectamine (6.5 = 10^3^ mean fluorescence) and N‐TER (1.2 = 10^3^ mean fluorescence) (see Figure 2a and also Figure S4a in the Supporting Information). Concerning neuronal cells, there were no significant differences between P4, Lipofectamine, and N‐TER (Figure 2b; Figure S4b, Supporting Information). The viability assay showed that after 24 h of incubation with P4 particles, the survival of astroglial and neuronal cells was not significantly affected (88% ± 5.5% and 89% ± 7%, respectively). On the other hand, decreased levels of cell survival were detected for Lipofectamine (glial cells: 78.4% ± 8.5% and neurons: 65.4% ± 4%) and N‐TER transfection (glial cells: 81% ± 6%, neurons: 44% ± 19%) with respect to a buffer control (glial cells: 94% ± 1.7% and neurons: 98.5% ± 0.6%) (see Figure 2c,d). To investigate whether endocytic pathways were involved in the uptake of particles, we preincubated cells with inhibitors of three endocytic pathways (chlorpromazine, an inhibitor of clathrin‐mediated intracellular transport; filipin, an inhibitor of cholesterol‐dependent caveolar cell transport; and amiloride, an inhibitor of Na^+^/H^+^ exchange in ligand‐independent, nonselective transport by macropinocytosis) before transfection (Figure 2e,h). We found that Amiloride led to the strongest reduction of uptake of siRNA both in glial (77.5% ± 6.4%) and neuronal (80% ± 9.1%) cell cultures. However, the combination of two inhibitors was needed to completely stop siRNA uptake, suggesting that more than one endocytic route is involved in the particle uptake by the cells. The internalization of siRNA using the P4 supramolecular particles was also confirmed by visualizing the siRNA–Alexa488 inside the cells using confocal microscopy (Figure 2f,i). Moreover, we quantified the colocalization of siRNA–Alexa488 with acidic endosomes and lysosomes stained with Lysotracker using Pearson's coefficient (Supporting Information, Figure 2g,j). P4–siRNA particles internalized by glial cells and neurons showed less colocalization with acidic endosomes and lysosomes (glial cells: 0.53 ± 0.04, neurons: 0.45 ± 0.14) relative to Lipofectamine (glial cells: 0.59 ± 0.1, neurons: 0.69 ± 0.14), indicating that siRNA is reaching the cytoplasm. N‐TER showed higher colocalization in glial cells (0.89 ± 0.1) indicating that siRNA is not escaping from the lysosomes; however, neuronal cells that survived transfection (44%) showed less colocalization (0.32 ± 0.2) than P4–siRNA particles.

**Figure 2:**
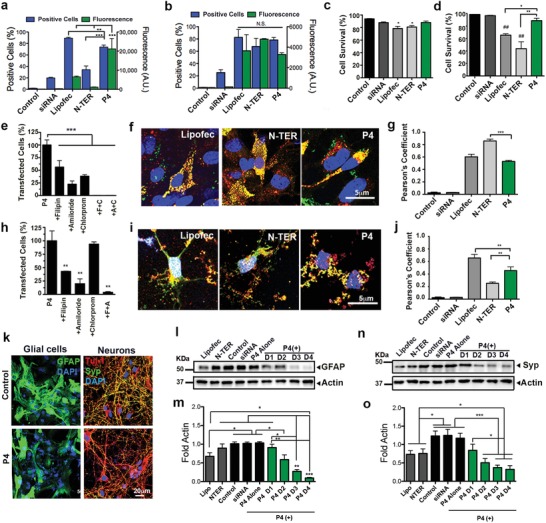
siRNA transfection and protein knockdown of neural cells. a,b) Percentage of transfected cells (blue graph) and amount of internalized fluorescently labeled siRNA–Alexa488 per cell (green graph) in a) astroglial and b) neuronal cell cultures. c,d) Percentage of cell survival in c) astroglial and d) neuronal cell cultures 24 h after transfection. e,h) Percentage of e) astroglial and h) neuronal cells transfected with P4 particles after their preincubation with inhibitors of three endocytic pathways: chlorpromazine (chlorprom or C), an inhibitor of clathrin‐mediated intracellular transport; filipin (F), an inhibitor of cholesterol‐dependent caveolar cell transport; and amiloride (A), an inhibitor of Na^+^/H^+^ exchange in ligand‐independent, nonselective transport by macropinocytosis. f,i) Confocal images of f) astroglial i) and neuronal cells stained with Lysotracker (red, endosomes), siRNA–Alexa488 (green, siRNA), and 4′,6‐diamidino‐2‐phenylindole (DAPI) (blue, nucleus) 24 h post‐transfection. g,j) Graph representing the Pearson's coefficient for siRNA/Lysotracker colocalization in g) astroglial and j) neuronal cell culture (−1 ≤ Rr ≤ 1, where 1 = maximum colocalization and −1 = maximal exclusion). k) Confocal images of astroglial cells stained with GFAP (green) and neuronal cells stained with synaptophysin (Syp, green), Tuj‐1 (red), and DAPI (blue) 24 h after transfection. l–o) Western blots and densitometry (intensity values normalized to actin) of l,m) GFAP and n,o) synaptophysin marker in neural cultures transfected with different P4 doses (doses 1, 2, 3 and 4 have concentrations of P4 supramolecular particles of 34 × 10^−6^, 52 × 10^−6^, 78 × 10^−6^, and 104 × 10^−6^ m, respectively, and of 65 × 10^−9^, 100 × 10^−9^, 150 × 10^−9^, and 200 × 10^−9^ m for siRNA, respectively) 24 h post transfection. *P < 0.05, ** *P* < 0.01, and **P < 0.001, least significant difference test (LSD) test (*n* = 7). All the experiments were performed using a dose 4 (P4: 104 × 10^−6^ m, siRNA: 200 × 10^−9^ m) except for western Blot analysis where four doses were used.

In order to demonstrate the ability to downregulate protein expression using P4 particles, we selected proteins that did not compromise cell viability such as glial fibrillary acidic protein (GFAP) and synaptophysin. We specifically selected GFAP, an intermediate filament expressed by astroglial cells in the CNS, since it plays a key role in astrocyte–neuron communication and it is upregulated in dementia, inflammatory demyelinating diseases, acute traumatic brain injury, and neurodegenerative diseases such as Alzheimer's disease, among others.^19^, ^20^ The selection of synaptophysin was motivated by the extensive presence of this synaptic vesicle (SV) glycoprotein amounting to 10% of SV proteins in the CNS.^21^ In addition, previous molecular studies have hinted at a number of diverse roles for synaptophysin in synaptic function including exocytosis, synapse formation, biogenesis, and endocytosis of synaptic vesicles.^21^, ^22^, ^23^

Our strategy was to transfect neural cells with P4 particles carrying the positive siRNA for GFAP or synaptophysin for astroglial or neuronal cell cultures, respectively. After incubation for 24 h with P4 particles carrying the positive siRNA (P4 (+)), western blot analysis showed a knockdown of GFAP and synaptophysin expression in astroglial (see Figure 2k–m and also Figure S5a,b in the Supporting Information) and neuronal cells, respectively (see Figure 2k,n,o and also Figure S5d,e in the Supporting Information). Neuronal cell cultures were also stained with the neuronal marker β‐III tubullin (Tuj‐1, see Figure 2k). Neural cells transfected with four increasing doses of P4–siRNA (dose 1: 34 × 10^−6^ –65 × 10^−9^ m; dose 2: 52 × 10^−6^ –100 × 10^−9^ m; dose 3: 78 × 10^−6^ –150 × 10^−9^ m; dose 4: 104 × 10^−6^ –200 × 10^−9^ m) showed × a dose‐dependent knockdown of GFAP and synaptophysin (see Figure 2l–o and the Supporting Information). In both cases, the highest dose (dose 4: 104 × 10^−6^ m for P4 and 200 × 10^−9^ m for siRNA) showed a significant decrease in protein expression by western blot and immunocytochemistry. Lipofectamine and N‐TER (concentration as suggested by provider and 200 × 10^−9^ m for siRNA) showed a decrease in protein expression with respect to control conditions but they still remained higher than P4 (+) at doses 2–4 (Figure 2m,o).

The dose‐dependent downregulation of both genes did not affect neural cell survival, which maintained values of 92.2% ± 1.4% for glial cells and 90% ± 2% for neuronal cells at dose 4 (Figure S5c,f, Supporting Information). Peptide alone (P4) and P4 carrying a negative control siRNA (P4 (−)) (siRNA at 65 × 10^−9^ and 200 × 10^−9^ m equivalent to doses 1 and 4) and free positive siRNA (siRNA at 200 × 10^−9^ m, equivalent to dose 2) showed similar protein levels to the control conditions, indicating the absence of nonspecific silencing by the peptide (Figure 2l–o; Figure S5a,b,d,e, Supporting Information).

A time course of the effect of siRNA at dose 4 (siRNA at 200 × 10^−9^ m) on protein levels showed that the highest protein knockdown of 83% happens between 24 and 48 h and cells, started to re‐express the proteins after 72 h of transfection without affecting cell survival (see Figure S6g–l in the Supporting Information). Our system exhibits one of the highest protein silencing efficacies compared to other similar ones reported in the literature (Table S3, Supporting Information).

We conjugated several bioactive sequences to P4 to demonstrate the possibility of functionalization without affecting the biophysical properties of the peptide (**Figure** **3**a; Table S1, Supporting Information). We synthesized P4 derivatives carrying arginyl‐glycyl‐aspartic acid (RGD) for integrin binding (P4–RGD) or hexa‐histidine (P4–His6) to facilitate endosomal escape.^24^, ^25^ The P4 derivatives were found to have similar binding behavior for siRNA compared to peptide P4 (see Figure 3b). Moreover, other biophysical properties were not found to change upon functionalization, including the structural transition upon siRNA binding (Figure 3c; Figure S6a,b, Supporting Information), hydrodynamic diameter (Figure 3d), and ζ‐potential (Figure 3e). Similarly to nonfunctionalized peptide P4, we found that the maximum change in secondary structure peaked at a peptide‐to‐siRNA ratio of 12:1 (Figure S6c, Supporting Information). According to DLS data, the hydrodynamic diameters of functionalized peptides increased from 20–50 to 400–450 nm after adding siRNA (Figure 3d). This was verified using SAXS data with a polydisperse sphere model, which yields particle diameters on the order of 425 nm (Figure S6d and Table S2, Supporting Information); this was also confirmed by nanoparticle tracking analysis (Figure S6e, Supporting Information). A possible strategy to reduce and homogenize the size of the particles in order to facilitate translation to in vivo applications could involve the addition of polyethylene glycol (PEG) to peptides for enhanced colloidal stability.^26^ Functionalized peptides had slightly positive ζ‐potential, P4–His6: +5.1 and +4.9 mV, and P4–RGD: +7.1 and 4.7 mV before and after binding to siRNA, respectively (Figure 3e). These values are similar to those obtained for P4 (7.3 and 4.7 mV before and after siRNA binding) suggesting that the surface of the particles have similar charge densities after functionalization. In addition, by cryo‐TEM and AFM imaging we confirmed particle formation in the functionalized peptides (Figure 3f; Figure S6f–i, Supporting Information).

**Figure 3:**
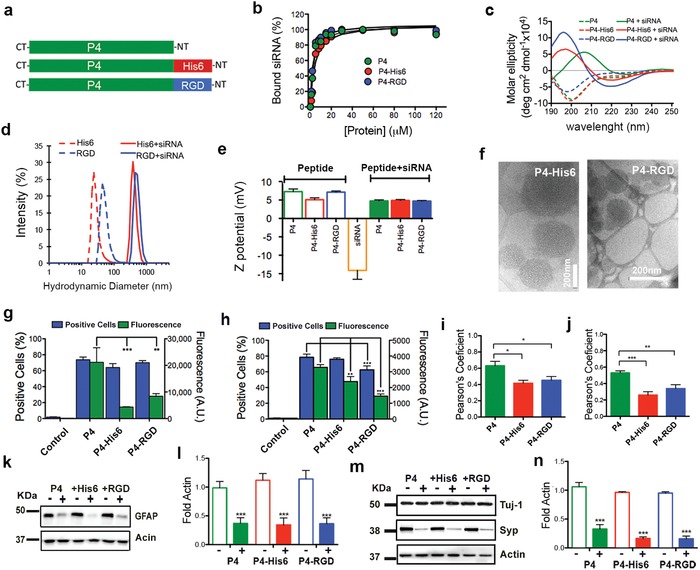
Functionalization of P4 supramolecular particles. a) Schematic representation of P4 peptide functionalized with bioactive peptides His6 (red) and RGD (blue). b) Plot of percentage of bound siRNA by P4, P4–RGD, and P4–His6 peptides calculated from a gel electrophoresis assay. c) Circular dichroism of P4, P4–His6, and P4–RGD peptides alone (dashed lines) and with siRNA (solid lines). d) Hydrodynamic size of P4, P4–His6, and P4–RGD peptides alone (dashed lines) and with siRNA (solid lines). e) ζ‐potential of peptides alone (empty bars) and with siRNA (solid bars) (results of P4 taken from Figure 1 are included for comparison purposes). f) Cryo‐TEM of peptide particles coassembled with siRNA. g,h) Percentage of positive transfected cells (blue graphs) and amount of internalized fluorescently labeled siRNA–Alexa488 per cell (green graph) with functionalized peptide P4 particles in g) astroglial cell and h) neuronal culture. i,j) Graph showing the Pearson's coefficient values for siRNA/Lysotracker colocalization in i) astroglial and j) neuronal cell culture (−1 ≤ Rr ≤ 1, where 1 = maximum colocalization and −1 = maximal exclusion). k–n) Western blots and densitometry (intensity values normalized to actin) of k,l) GFAP marker and m,n) synaptophysin (Syp) after 24 h of transfection in P4 functionalized with His6 (P4–His6) and RGD (P4–RGD) with a negative (−) or positive (+) siRNA. *P = 0.05, **P = 0.001, LSD test, *n* = 8 (GFAP) and *n* = 6 (synaptophysin). All the experiments were performed using a dose 4 (P4: 104 × 10^−6^ m, siRNA: 200 × 10^−9^ m).

Our in vitro studies showed that a similar percentage of glial and neuronal cells were transfected with P4–His6 (64.1%, 76%) and P4–RGD (70%, 62.5%), as we had observed with P4 peptide (73.8%, 78.5%) (see Figure 3g,h). However, we found that fluorescent siRNA–Alexa488 internalized by neural cells was statistically lower in functionalized P4–His6 (glial cells: 4.4 × 10^3^, neurons: 2.4 × 10^3^) and P4–RGD (glial cells: 8.4 × 10^3^, neurons: 1.4 × 10^3^) relative to P4 supramolecular particles (glial cells: 21.2 × 10^3^, neurons: 3.8 × 10^3^) (Figure 3g,h; Figure S4c,d, Supporting Information). These results indicate that less functionalized particles were internalized by the same number of transfected neural cells. We also found from measurements of the Pearson's coefficient (colocalization between labeled siRNA and lysotracker) that more siRNA escapes into the cytoplasm in neural cells transfected by functionalized particles compared to P4 particles (Figure 3i,j). Furthermore, cells transfected with P4–His6 and P4–RGD showed similar knockdown of GFAP and synaptophysin as with P4 peptide transfection (Figure 3k–n). These results suggest that functionalized P4 particles are more efficient than P4 particles since they can knock down GFAP and synaptophysin in a similar way as P4 using a smaller number of particles. Finally, nonfunctionalized particles do not significantly affect cell behavior and survival (Figure S7, Supporting Information). Hence, we showed that functionalization with bioactive sequences is possible without disrupting the capacity of P4 peptide to form particles. In addition, functionalization could be a way to provide cell recognition and enhance endosome escape, leading to more efficient target protein knockdown in neural cells.

We carried out electrophysiological studies in neuronal culture to determine the feasibility of using the supramolecular particles to perform functional protein screening. After 15–20 days in culture, cortical neurons, stained with the neuronal marker Tuj‐1, connect to each other with active synapses, forming a network of neurites and displaying spontaneous electrophysiological activity. We hypothesized that the observed large reduction in synaptophysin (**Figure** **4**a) could induce a significant functional change in electrical activity after transfection with the particles carrying synaptophysin–siRNA. In order to investigate this possibility, we used multielectrode arrays (MEA) to assess the spontaneous and synchronized activity of cortical neuronal cultures over time as illustrated in the spike raster plots at day 0 and day 3 from a control treated well (Figure 4b,c; Figure S8a–c, Supporting Information). We found that neuronal cells transfected twice within a period of 10 days (transfection 1 at day 0 and transfection 2 at day 6) with P4, P4–His6, and P4–RGD particles showed reduced spontaneous and synchronized activity relative to cultures exposed to buffer or negative siRNA (Figure 4d,e). On cells transfected with Lipofectamine and N‐TER, cell survival was reduced to 20% (Figure S8d, Supporting Information) making it difficult to analyze electrical activity. None of the P4 particles affected cell survival significantly (cell survival remained above 70%) after the two transfections as established by flow cytometry, immediately following MEA recordings (day 10) (Figure S8d, Supporting Information). Moreover, synchronized activity (see Supporting information for definition) was reduced significantly 3 days post transfection (Figure S8e,f, Supporting Information). These results demonstrate that reduction in synaptophysin using the different supramolecular particles has a direct effect on synchronous network activity as well as in spike and burst frequency in primary neuronal cultures.

**Figure 4:**
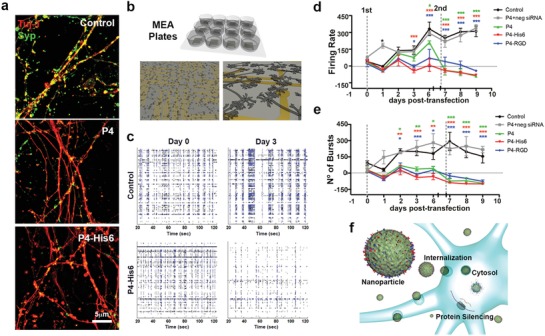
Electrophysiological studies of functional gene knockdown of neuronal cell cultures by supramolecular particles. a) Structured illumination microscopy (SIM) images of cortical neuronal cells stained with Tuj‐1 (red) and synaptophysin (Syp, green) 24 h after transfection. b) Concept scheme of multielectrode array plates (MEA plates) for neural cell recording. c) Raster plots of a single MEA plate well of representative control and P4–His6‐treated cells before transfection (day 0) and 72 h post‐transfection (day 3). Each line represents the signals detected by a single electrode of the MEA array, during 100 s of recordings. d) Longitudinal progression of spike firing frequency and e) burst number. f) Schematic representation of cells transfected by P4 particles. Two‐way repeated measures one‐way analysis of variance (ANOVA) were performed for firing frequency and burst number. *P = 0.05, **P = 0.001, and ***P = 0.0001. All the experiments were performed using a dose 4 (P4: 104 × 10^−6^ m, siRNA: 200 × 10^−9^ m).

In conclusion, we have developed supramolecular particles containing designed peptides capable of encapsulating siRNA with the capacity to promote molecular aggregation. These supramolecular particles transfect and knock down efficiently protein expression in glial and neuronal cell cultures without significant toxicity. The easily prepared peptide particles can be internalized by neural cells, deliver siRNA cargo into the cytoplasm, and silence efficiently protein expression (Figure 4f). The supramolecular systems described above offer a strategy to build single‐component peptide particles with high transfection efficiency and downregulation of target proteins potentially useful for discovery of therapeutic targets.

## Conflict of Interest

The authors declare no conflict of interest.

## Supporting information

Supplementary
