# Supplementary material for: Peptide–siRNA Supramolecular Particles for Neural Cell Transfection
Source: Adv Sci (Weinh). 2018 Dec 5;6(3):1801458. doi: 10.1002/advs.201801458 (PMC6364495; doi:10.1002/advs.201801458)
Supplement: Supplementary file 1 — Supplementary [file ADVS-6-1801458-s001.pdf]

Copyright WILEY-VCH Verlag GmbH & Co. KGaA, 69469 Weinheim, Germany, 2018.

Supporting Information

for *Adv. Sci.,* DOI: 10.1002/advs.201801458

Peptide–siRNA Supramolecular Particles for Neural Cell Transfection

*Armando Hernandez-Garcia, Zaida Álvarez, Dina Simkin, Ashwin Madhan, Eloise Pariset, Faifan Tantakitti, Oscarde J. Vargas-Dorantes, Sungsoo S. Lee, Evangelos Kiskinis, and Samuel I. Stupp**

Supporting Information

Peptide-siRNA supramolecular particles for neural cell transfection

*Armando Hernandez-Garcia†, Zaida Álvarez†, Dina Simkin, Ashwin Madhan, Eloise Pariset, Faifan Tantakitti, Oscar de J. Vargas-Dorantes, Sungsoo S. Lee, Evangelos Kiskinis, and Samuel I. Stupp**

Dr. A. Hernandez-Garcia, Dr. Z. Álvarez, A. Madhan, E. Pariset, Dr. F. Tantakitti, Dr. S. S. Lee, Prof. S. I. Stupp
Simpson Querrey Institute, Northwestern University, Chicago, IL, 60611, USA

Dr. A. Hernandez-Garcia and O. de J. Vargas-Dorantes
Department of Chemistry of Biomacromolecules, Institute of Chemistry, National Autonomous University of Mexico, Ciudad Universitaria, Mexico City, 04510, Mexico.

Dr. D. Simkin
Department of Pharmacology, Feinberg School of Medicine, Northwestern University, Chicago, IL, 60611, USA

Dr. D. Simkin and Prof. E. Kiskinis
The Ken & Ruth Davee Department of Neurology & Clinical Neurological Sciences, Department of Physiology, Feinberg School of Medicine, Northwestern University, Chicago, IL 60611, USA

Prof. S. I. Stupp
Department of Chemistry, Northwestern University, Evanston, IL 60208, USA
Department of Materials Science and Engineering, Northwestern University, Evanston, IL 60208, USA
Department of Biomedical Engineering, Northwestern University, Evanston, IL 60208, USA
Department of Medicine, Northwestern University, Chicago, IL 60611, USA

†These authors contributed equally to this work.

*Corresponding author: S.I. Stupp. e-mail: s-stupp@northwestern.edu.

Keywords: neurons, glial cells, transfection, supramolecular particles, protein engineering, GFAP, synaptophysin, knockdown.

Materials

All reagents were purchased from Sigma-Aldrich and used without any further purification unless otherwise indicated. HEPES sodium salt was purchased from EMD chemicals (Merk, Germany). Novex Sharp Pre-stained protein standard, O'RangeRuler 5bp DNA ladder ready-to-use, NoLimits 25bp DNA fragment and Lysotracker® Red DND-99 were purchased from Thermo Fisher Scientific. Silencer® GFP (eGFP) siRNA, silencer® Negative Control siRNA #1 and Negative Control 1 GFP siRNA were purchased from Ambion (Thermo Fisher Scientific), GFAP siRNA (mouse) sc-35466 from Santa Cruz Biotechnology and SypMSS277527 Stealth RNAi from Invitrogen. Alexa Fluor 488 conjugated siRNA was synthesized by Integrated DNA Technologies. Transfection systems N-TER peptide nanoparticle siRNA was acquired from Sigma-Aldrich and Lipofectamine® RNAiMAX from Thermo Fisher Scientific.

Peptide Synthesis

The peptides were synthesized in the Peptide Synthesis Core at the Simpson Querrey Institute for BioNanotechnology of Northwestern University using a CEM Liberty microwave-assisted peptide synthesizer. Standard fluoren-9-ylmethoxycarbonyl (Fmoc) solid-phase peptide synthesis was used on low loading (~0.3-0.4 mmol/g) pre-loaded Wang resin (100-200 mesh). For each coupling, 5 equivalents of Fmoc- protected amino acid in DMF was added with 5 equivalents of *N,N,N′,N′*-tetramethyl-O-(1H-benzotriazol-1-yl)uronium hexafluorophosphate (HBTU) in DMF and 10 equiv of *N,N*-diisopropylethylamine (DIPEA) in NMP. Fmoc removal was accomplished using a solution of 20% piperidine in DMF and 0.1 M 1-hydroxybenzotriazole (HOBt). Peptides were cleaved from the resin using a mixture of 95% trifluoroacetic acid (TFA), 2.5% water, and 2.5% triisopropylsilane for 3 h. Crude peptide was precipitated from this solution using cold diethyl ether.

Purification was carried out on a reversed-phase high-performance liquid chromatography (HPLC, Shimadzu Prominence) using a C18 preparative column (Agilent PLRPS, dimension 21x150 mm, 100 Å pore size). Peptides were eluted using a water/acetonitrile gradient with 0.1% TFA solvent system with 2% acetonitrile as a starting condition (5 min). The water/acetonitrile gradient conditions for purification of P4 and P4-His6 were: a first linear ramp from 98:2 to 26:74 during 5 min and a second linear ramp to 34:66 during 50 min. For P4-RGD the final gradients were: 25:75 and 35:65 for the first and second ramp, respectively. All the peptide fractions were the last eluted peak at the second ramp. All the peptides started to elute around gradient 30:70 (min 30 for P4 and P4-His6, while min 37 for P4-RGD). For better purity peptides could be re-purified with the same method for a second time. Peptide purity was determined by HPLC (Agilent 1260 Infinity, eluted during 30 minutes with a gradient of water: acetonitrile from 95:5 to 5:95 in 0.1% TFA) and by electrospray ionization mass spectrometry in positive mode (ESI-MS, Agilent 6510 Q-TOF). Peptide solutions were lyophilized and stored at -20 °C in a sealed polypropylene tube until further use.

Peptide-siRNA preparation

Peptide-siRNA aqueous samples in 25 mM pH 7.4 HEPES buffer were prepared by diluting aliquots taken from peptide (usually 470 μM) and siRNA (50 μM) stock solutions and incubated at room temperature overnight. Stock solutions were prepared by dissolving in Milli-Q water an amount of lyophilized peptide and vortexing by 0.5-1 minute. Peptides were weighted out using an analytic scale (Mettler Toledo) with 0.001 mg sensitivity. Sterile peptides were prepared by irradiating the weighted lyophilized powder for 90 minutes with UV light using a safety hood.

Electrophoresis Gel

10 μL of peptide-siRNA sample (siRNA final concentration was 200 nM) were mixed with 2 μL of 6x loading buffer and immediately 10 μL were loaded into Novex® TBE 6% polyacrylamide Gels with 1 mm thickness (Thermo Fisher Scientific) and run on the XCell *SureLock*® Mini-Cell at 100V for 50 min at 4 C with 1X Novex® TBE Running Buffer. siRNA in the gel was stained for 20 min with SYBR® Gold Nucleic Acid Gel Stain (Thermo Fisher Scientific) and imaging using a gel imaging FluorChem E (ProteinSimple). The band intensities of the gels were analyzed and compared using ImageJ software.

Circular Dichroism

Ellipticity θ of 30 μM peptide solutions in 0.5 mM pH 7.4 HEPES buffer mixed with different concentration of siRNA were measured using a 0.1 cm quartz cuvette using a Jasco J-815 Circular Dichroism spectrophotometer. Every measurement was the average of 5-7 scans taken between 260-190 nm range using standard sensitivity at a scanning speed of 100 nm per min with the following parameters: DIT: 2 seconds, band width: 2 nm and data pitch: 1 nm. Background ellipticity of corresponding siRNA concentration in in HEPES buffer was removed in every sample. Molar ellipticity was calculated using the following equation:

$$[\theta] = \frac{\theta \times 100 \times M}{C \times l \times n}$$

where the ellipticity θ is in degrees, M is the molecular mass of the peptide, C is the peptide concentration in mg/mL, L is the optical path length in cm and n is the number of amino acid residues in the peptide.

Dynamic Light Scattering and Zeta Potential

The measurements were done using a Zetasizer-Nano equipment (Malvern). The hydrodynamic radius was the average of 5-7 measurements (12 runs for 10 seconds) measured at 25 C using a low volume quartz cuvette. The sample solutions were dissolved in 25 mM pH

7.4 HEPES buffer with final concentrations of 450 µM and 0.8 µM for the peptide and siRNA, respectively. The zeta potential measurements were taken in automatic mode at 25 C with a pause of 2-3 min between every measurement to avoid warming up. Between 100-200 µL of peptide-siRNA sample dissolved in 10 mM, pH 7.4 phosphate buffer were deposited at the bottom of a disposable folded capillary cell DTS1070 filled up with buffer.

Cryogenic Transmission Electron Microscopy (Cryo-TEM)

It was performed using a JEOL 1230 TEM fitted with a LaB6 filament, which was operated at an accelerating voltage of 100 kV. Samples were prepared using a Vitrobot Mark IV (FEI) vitrification robot operating at 25 °C with 95-100% humidity. The solution (6.5 µL) was deposited on a 300 mesh copper grid with lacey carbon or QUANTIFOIL®–Holey carbon support (Electron Microscopy Sciences), blotted, and plunged into a liquid ethane reservoir cooled by liquid nitrogen. For imaging, the vitrified samples were transferred to a Gatan 626 cryo-holder under liquid nitrogen. A large area was scanned to observe the overall structures and images were acquired using a Gatan 831 bottom-mounted CCD camera.

Atomic Force Microscopy (AFM)

Approximately 10-20 µL of sample were deposited on silica wafer substrate previously plasma irradiated for 15 min. After 5 minutes the substrate was rinsed with 1 mL of MQ-water and slowly dried using nitrogen blow. Any excess of liquid was removed using a tissue paper. Nanoparticles were imaged using an Dimension Icon AFM (Bruker) in tapping mode and images were analyzing using the software Image SXM 197 version 1.62.

Stability of P4-siRNA particles in different media

P4 peptide and siRNA were mixed in 25 mM HEPES at pH 7.4 to a final concentration of 280 µM and 0.8 µM, respectively, and incubated overnight. Freshly prepared DMEM (Invitrogen®), FBS (Gibco®) or DMEM supplemented with 10% FBS was added to particles in solution and incubated at room temperature. Aliquots of 20 µL were taken and kept frozen after different incubation times. Controls with naked siRNA were also prepared. Samples were run in a native 6% polyacrylamide gel (Sigma Aldrich). 10 µL of P4-siRNA aliquot was loaded into the gel with 2 µL of 6x DNA Loading Dye (Thermo Fisher Scientific). An electrophoresis was run on the Mini PROTEAN® Tetra Cell at 100 V (14.28 V/cm) for 40 min in TBE 1x Running Buffer (Thermo Fisher Scientific). Gels were stained for 20 min with SYBR® Green I nucleic acid stain (Thermo Fisher Scientific) and imaged with Azure c300 (Azure Biosystems).

Stability of P4-siRNA particles in the presence of Heparin Sulfate

P4-siRNA particles were prepared as described in a previous section. For this assay, 5 µL of particles in aqueous solution were mixed with 50 mg/mL of heparin sulfate solution

(Inhepar®, 5000 U/mL equivalent to) and Milli-Q® water to a final concentration of 0.01 mg/mL (1 U/mL) in a final volume of 10 μL. Samples were incubated at room temperature for 2h. After incubation, samples were run in a native 6% polyacrylamide electrophoresis gel as described in the section above.

<u>DNAse I Digestion Test</u>

A solution of 40 μM peptide and 0.2 μM DNA dissolved in 10X DNAse I buffer (Invitrogen) was mixed with DNAse I (Invitrogen) for a final enzyme concentration of 0.003 U/μL. The solution was incubated at 37 C and at each time point, 12 μL were mixed with 2.7 μL of 5X TBE buffer in ice to quench the enzyme. Then an aliquot of 4 M NaCl was added to every time point sample to a final concentration of 300 μM after incubation for 3 days at 4°C the samples were ran in an electrophoresis gel.

<u>Proteinase K Digestion Test</u>

A previously prepared solution of 50 μM peptide and 0.2 μM siRNA dissolved in buffer 25 mM HEPES pH 7.4 were incubated during 24h at 37C in presence of Proteinase K from *Tritirachium album* (Sigma-Aldrich). After this time an aliquot of it was ran in an electrophoresis gel as mentioned above.

<u>Salt stability of P4-siRNA particles</u>

Sample solutions of 70 μM P4 peptide and 0.2 μM of siRNA in 25 mM HEPES pH 7.4 were incubated with different concentrations of NaCl (0-300 mM) for 24h at room temperature. The samples were run in a native 6% polyacrylamide electrophoresis gel prepared manually.

<u>Stability of P4-siRNA particles with different media</u>

P4-siRNA particles ([P4] = 235 μM and [siRNA] = 2 μM]) in 25 mM HEPES pH 7.4 were mixed 1:1 with DMEM or DMEM-FBS 20% and measurements of hydrodynamic radius were done by DLS as described before.

<u>Animals</u>

All animal housing and procedures were performed in accordance with the Public Health Service Policy on Humane Care and Use of Laboratory Animals and all procedures were approved by the Northwestern University Institutional Animal Care and Use Committee.

<u>Primary Cultures</u>

Glial cells were derived from the cerebral cortex of newborn mice (P0), as described[1]. Briefly, cerebral cortices were dissected free of meninges in dissection buffer (PBS 0.6% glucose (Sigma-Aldrich), 0.3% BSA (Sigma-Aldrich)) and digested with trypsin (Biological Industries) and DNAse I (Sigma-Aldrich) for 10 min at 37° C. The tissue was dissociated in

Dulbecco's Modified Eagle Medium (DMEM, Biological Industries), 10% normal horse serum (NHS, GIBCO), 1% penicillin-streptomycin (Pen-Strep, Biological Industries) and 2mM L-glutamine (Biological Industries). After centrifugation and re-suspension, cells were plated and grown to confluence at 37°C, 5% CO_2(approximately 25-30 days *in vitro (div)*). All the experiments were performed using glial cells from the first passage (Ps1). Passage 1 (Ps1) cells were cultured at a density of $2x10^5$ cells/cm2 for 15 days *in vitro* (*div*) in DMEM, 1% Pen-Strep and 2mM L-glutamine on non-coated culture plastic (for Western blotting, flow cytometry) or glass (for immunocytochemistry). The cell composition and biochemical characterization of control and reference glial conditions have been described previously[2].
Neurons were obtained from embryonic brains, as described[3]. Briefly, a time pregnant mouse was sacrificed by cervical dislocation and the embryos were extracted at embryonic day 16 (E16). Cerebral cortices were dissected free of meninges in a solution of PBS with 0.6% glucose (Sigma-Aldrich), 0.3% BSA (Sigma-Aldrich) and digested with trypsin (Biological Industries) DNAse I (Sigma-Aldrich) for 10 min at 37ºC. The tissue was mechanically dissociated, centrifuged and resuspended in CO_2-equilibrated DMEM supplemented with 10% NHS, 1% Pen-Strep, 0.5mM L-Glutamine, 5.8µl/ml $NaHCO_3$ (Sigma-Aldrich). The cell suspension was pre-plated at 37ºC. After 30 min, the supernatant was collected, centrifuged (1000g 5min), resuspended in neuronal culture medium (NB, 1%NHS, 1% Pen-Strep, 0.5mM L-glutamine, 22 µM glutamic acid (Sigma-Aldrich), 2% B27 (Gibco), 5.8µl/ml $NaHCO_3$) and plated at a density of $2.5x10^5$ cells/cm2 directly on poly-D-lysine (Sigma-Aldrich) coated tissue culture plate (for Western blotting) or glass (for immunocytochemistry, flow cytometry) during 15*div.* Half-cell media was changed every 3-4 days.

Neural cell transfection

After 15div, P0 glial cultures and E16 primary neuronal cultures were transfected with GFAP-siRNA or Synaptophysin-siRNA (100 nM) encapsulated by the peptide (52 µM) in a serum-free medium during different time points (24h, 48h, 72h). Dose 1, 2, 3 and 4 of supramolecular particles have concentration of 34, 52, 78 & 104 μM for P4 and 65, 100, 150 and 200 nM for siRNA, respectively. The starvation media including Buffer (Control), naked siRNA (siRNA) were used as negative controls. The commercial positive controls Lipofectamine RNAimax (Lipofec) and N-TER were used as recommended by the producers. Negative siRNA were used to discard the effect on cellular response. The samples were either fixed in 4% PFA for immunocytochemistry or used for protein extraction and Western blot analysis.

Flow Cytometry and Cell Survival

Ps1 glial cells and Ps 0 embryonic neurons were cultured in 48-wells plates at a density of 2×10^5 cells/cm^2, and transfected with 100 nM fluorescently labeled siRNA-Alexa Fluor 488 encapsulated with different concentrations of peptide (explained above), Lipofectamine

RNAiMAX or N-TER prepared as suggested by the producers. 1-4 days post-transfection cells were washed carefully three times with 1x PBS. Each well was incubated with 200 μL trypsin until cells detached (did not exceed 10 min of incubation). Cells were then resuspended in 600 μL of media to neutralize the trypsin and were centrifuged for 15 min at 1.2 rpm. Supernatant was removed, and the pellet was re-suspended in approximately 40 µL of media before Flow Cytometry (BD LSRFortessa) was performed. DAPI (Sigma, 5 μg/mL) was added 1-3 min before measurements were taken to determine the absolute number of dead cells (excitation wavelength of 405 nm). Cell populations were gated based on cell size (FSC), granularity of the cytoplasm (SSC), multiple cells (FSC-W), UV fluorescence (DAPI filter). Percentage of positive cells was calculated from alive cells (DAPI negative). All the samples were measured in triplicates. Results were analyzed using FlowJo software.

Internalization Route

After 15 div neural cells were treated with either 5 µg/mL chlorpromazine hydrochloride, 1µg/mL fillipin III from *Streptomyces filipinensis*, 300 µM amiloride hydrochloride or a combination of them in fresh serum-free medium for 30 minutes before incubated with the peptide (52 µM) + siRNA-A488 (100 nM) for 15h. Samples were analyzed using flow cytometry as described in the previous section.

Western blot/ immunocytochemistry

For Western blot analysis, protein extracts were obtained from primary cultures after 1-3 div post transfection and total protein extracts were separated by SDS-polyacrylamide gel electrophoresis and electro-transferred to a nitrocellulose membrane (Bio-Rad). Membranes were blocked with 5% bovine serum albumin (BSA, Sigma-Aldrich) and incubated first with primary antibodies overnight at 4°C, and then with their corresponding secondary HRP-conjugated antibodies (1:3000; Santa Cruz Biotechnology). Protein signals were detected by the ECL chemiluminescent system (Amersham, GE Healthcare). Densitometry analysis, standardized to GADPH as a control for protein loading, was performed with ImageJ software (National Institutes of Health, USA). For quantification, triplicate samples were analyzed and at least three different experiments were used. For immunofluorescence fixed primary cultures samples were incubated with primary antibodies overnight at 4°C, and then with their appropriate Alexa 488 or Alexa 555 secondary antibodies (1:500, Molecular Probes). DAPI (1:500, Molecular Probes) was used to stain nuclei. Finally, the preparations were cover-slipped with Mowiol (Calbiochem) for imaging. The following primary antibodies were used for western blot and /or Immunocytochemistry; rabbit anti-GFAP (mature and reactive glial marker, 1:500-1:8000, Dako), mouse anti-Tuj-1 (neuronal marker 1:10000, Covance), mouse anti-Synaptophysin (pre synaptic vesicle protein, 1:200, Dako), and mouse anti-Actin (1:1000, Cell signaling).

Imaging/analysis of cells and co-localization

Fluorescent preparations were viewed and micrographs were captured with a Nikon A1R confocal laser-scanning microscope with GaAsP detectors and a Structured illumination Super-resolution microscope (SIM). Images were assembled in Adobe Photoshop (v. 7.0), with adjustments for contrast, brightness and color balance to obtain optimum visual reproduction of data. Morphometric, quantitative, live-image analyses were performed using ImageJ software (National Institutes of Health, USA). SiRNA and Lysotracker co-localization was quantified using the Intensity Correlation Analysis plug-in on pictures of siRNA stained with Alexa Fluor 488 and endosomes stained with Lysotracker (Thermo Fisher Scientific). This Pearson's correlation coefficient (Rr) measures the amount or degree of colocalization, and values range between 1 and −1, where 1 represent maximal co-localization and −1 maximal exclusion[4].

Electrophysiology- Multi electrode Array (MEA) Plates

12 well MEA plates with 64 electrodes per well were coated with PEI and laminin following Axion Biosystems instruction. Embryonic primary neurons were cultured during 15 days *in vitro*. During day 20 to day 30, 3 wells were used on each 12 well plate for each condition as well as 2 treatments (Dose 1 and 2) to reduce synaptophysin expression levels. Spontaneous network and synchronized activity was recorded using Axion Biosystems Maestro 768 channel amplifier and Axion Integrated Studios (AxIS) v2.3 software. The amplifier recorded from all channels simultaneously using a gain of 1200× and a sampling rate of 12.5 kHz/channel. After passing the signal through a Butterworth band-pass filter (300–5000 Hz) on-line spike detection (threshold = 6× *the* root-mean-square of noise on each channel) was done with the AxIS adaptive spike detector. All recordings were conducted at 37 °C with appropriate 5% CO_2/95% O_2. Spontaneous network activity was recorded for 2 min (baseline; D -1), and for 2 min each day after transfection. Active electrodes were defined as having >5 spikes/min and only wells with over 10 active electrodes during the baseline-recording period were used in the analysis. Synchronized activity was defined as spike and burst activity that occurred on 25% of the electrodes or more in a well within 100ms of each other. The mean burst number was used as a measure of neuronal activity as this demonstrates maturity of neuronal functional properties. All data reflects well-wide averages, with reported n's representing the number of wells per condition. The number of bursts following transfection treatment was expressed as a percent of baseline burst number from D-1 recordings for each treatment per well.

Statistics

Cell counts were expressed as mean cells/mm^2 ± standard deviation. Values were the average of three replicates of at least two different experiments. Data were analyzed by repeated measures ANOVA followed by *post-hoc* multiple comparison tests using StatView (SAS) program. Significance level was preset to $P < 0.05$. Data are expressed as means ± SEM.

Data availability

The data that support the findings of this study are available from the corresponding author upon reasonable request.

Supplementary Figures and Tables.

Table S1. Amino acid sequences of peptides.

Name	Sequence (from N- to C-terminal)	#amino acids	MW (kDa)
dsRBD*	MPVGSLQELAVQKGWRLPEYTVAQESGPPHKREFTITVRVEG-DBCO-N_3-TFVETGSGTSKQVAKRVAAEKLLTKFKT	70	8402
P1	MPVGSLQELAVQKGWRLPEYTVAQESGPPHKREFTITVRVE	41	4665
P2	TFVETGSGTSKQVAKRVAAEKLLTKFKT	28	3383
P3	SIRKLEYEIEELRLRIGGG	19	2231
P4	SIRKLEYEIEELRLRIGGGTFVETGSGTSKQVAKRVAAEKLLTKFKT	47	5240
P5**	AMLKAMLKAMAELMAKLY	18	2028
P6	MPVGSLQELAVQKGWRLPEYTVAQESGPPHKREFTITVRVEGGSIRKLEYEIEELRLRIG	60	6878
P7	AMLKAMLKAMAELMAKLY-PEG_{10}-TFVETGSGTSKQVAKRVAAEKLLTKFKT	46	5549
P4-His6	HHHHHHSIRKLEYEIEELRLRIGGGTFVETGSGTSKQVAKRVAAEKLLTKFKT	53	6063
P4-RGD	CRGDCSIRKLEYEIEELRLRIGGGTFVETGSGTSKQVAKRVAAEKLLTKFKT	52	5774

*Peptides are linked through a copper-free click chemistry reaction between dibenzocyclooctyne and azide group
**Sequence taken from Gellman S. H., et. al. JACS, 118(50), 1996, pp. 12487-12494

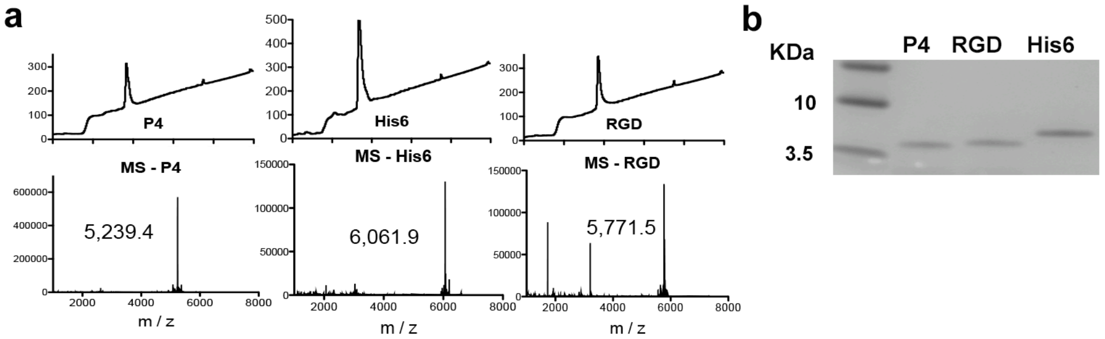

Figure S1. Analysis of purified peptides. (a) Chromatographs of purified peptides. **(b)** SDS-PAGE of purified peptides.

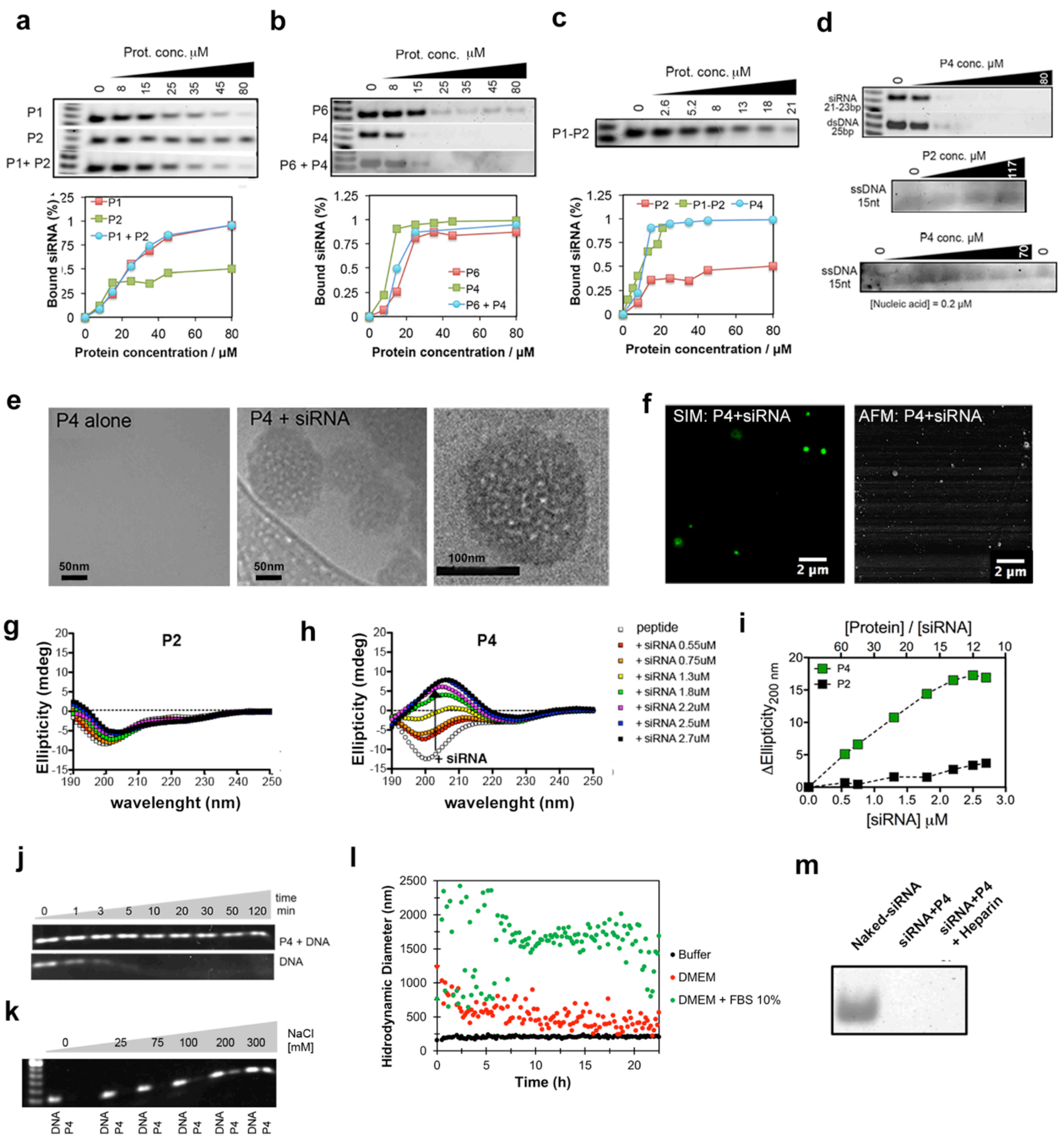

Figure S2. Binding properties of peptide P4-siRNA particles. Gel electrophoresis (top) and binding curve (bottom) of siRNA + **(a)** P1 & P2, **(b)** P6 & P4 and **(c)** P1-P2 (chemical analog of dsRBD). **(d)** Gel electrophoresis of P4 binding different nucleic acids: dsRNA and dsDNA (top gel), ssDNA (middle gel) and ssDNA (bottom gel). **(e)** Cryo-TEM images of P4 + siRNA particles and P4 alone. **(f)** Super-resolution Structured Illumination Microscopy (SIM) and Atomic Force Microscopy (AFM) of P4 + siRNA particles. **(g-h)** Circular dichroism of **(g)** P2 and **(h)** P4 with increasing concentrations of siRNA. **(i)** Plot of change in ellipticity of P4 with increasing concentrations of siRNA. **(j)** Electrophoresis gel of protection of P4-DNA particles against enzymatic DNAse attack. **(k)** Electrophoresis gel of salt stability of P4-DNA particles **(l)** P4-siRNA particle size incubated with HEPES buffer, DMEM media and DMEM+10% fetal bovine serum (FBS). **(m)** Electrophoresis gel of P4-siRNA particles incubated with 1U/mL (10µg/mL) of heparin sulphate for 2h at 25C.

Table S2. Peptide-siRNA particle sizes estimated by Small Angle X-ray Scattering (SAXS).

Sample (SAXS)	Diameter (nm)
P4	16.05
P4 + siRNA	508.02
P4-RGD	17.4
P4-RGD + siRNA	425.77

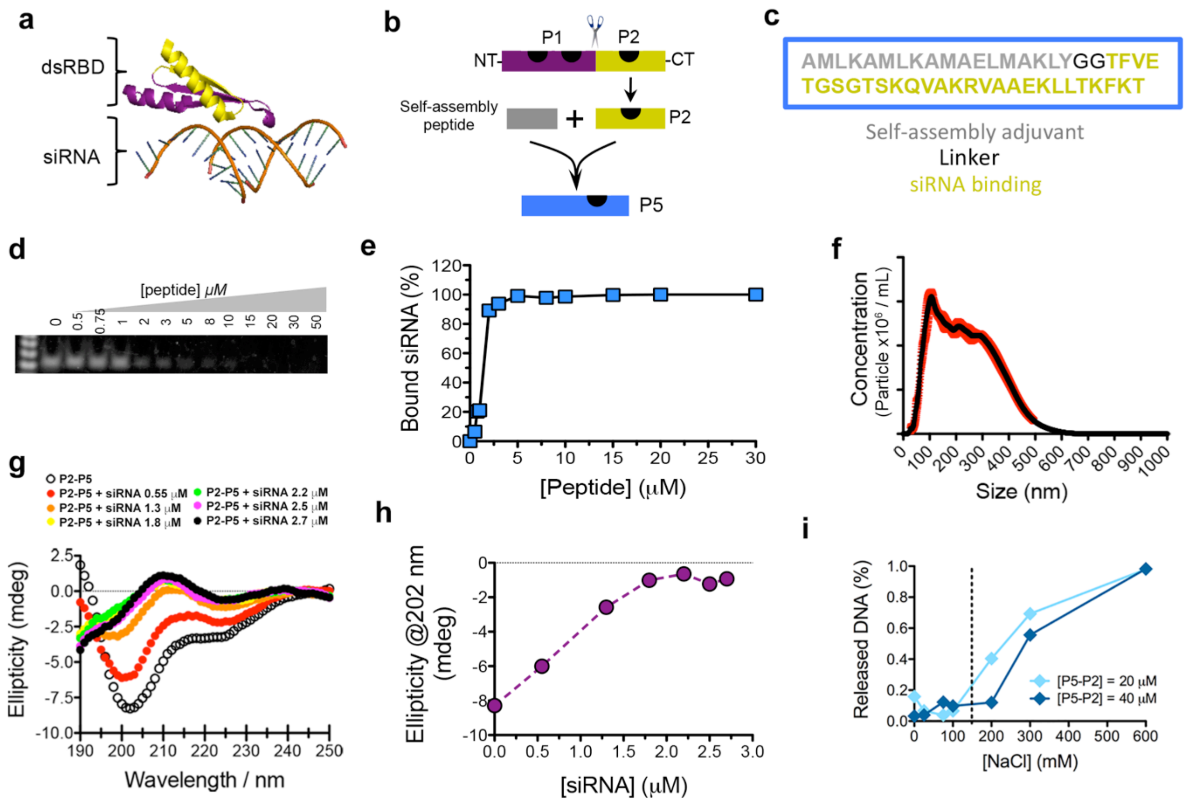

Figure S3. Design of modular peptide P2-P5 with amplified siRNA binding via self-assembly. (a) Scheme showing crystal structure of a dsRBD-binding domain from *X. laevis* binding siRNA. **(b)** C-terminus RNA binding motif P2 (in yellow) was extracted from the dsRBD and fused to a self-assembly enhancer peptide P5 (gray) to obtain P6. **(c)** Full sequence of the peptide P6. Below there is a short description of the function of each module. **(d)** Electrophoresis gel of peptide P6 bound to siRNA. **(e)** Binding plot derived from the electrophoresis gel. **(f)** Nanoparticle tracking analysis of P6 + siRNA. **(g)** Circular dichroism of P5 titrated with increasing concentrations of siRNA. **(h)** Plot showing the change in ellipticity of P6 titrated with siRNA. **(i)** Salt stability of P6-siRNA particles.

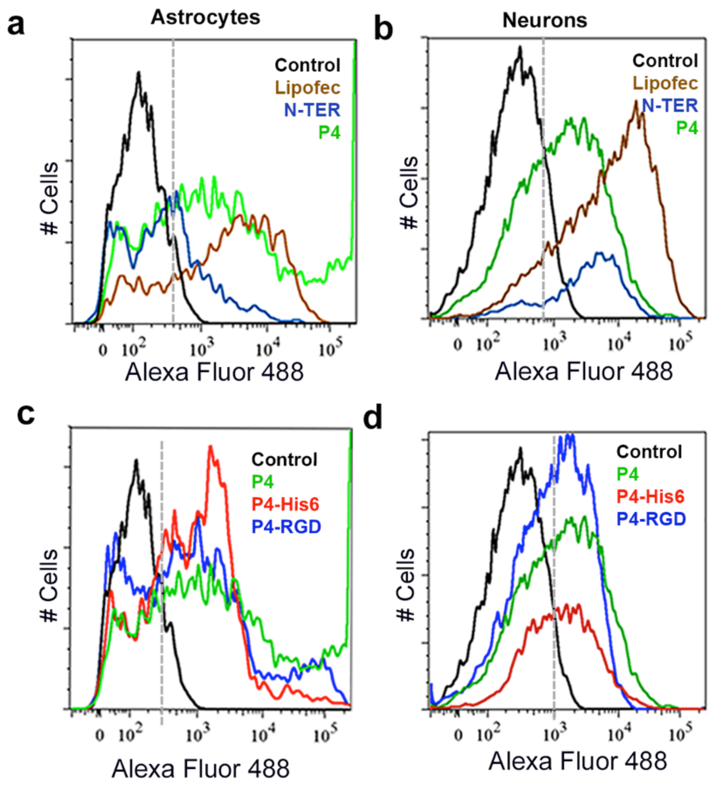

Figure S4. Flow cytometry histograms of neural cells transfected with peptide particles. (a,b) Histograms of **(a)** astrocytes and **(b)** neuronal cells transfected with starvation media (Control), Lipofectamine, N-TER and P4 + siRNA. **(c,d)** Histograms of **(c)** astrocytes and **(d)** neuronal cells transfected with functionalized P4-His6 and P4-RGD particles. Dose P4 particles-siRNA; 104 µM - 200 nM.

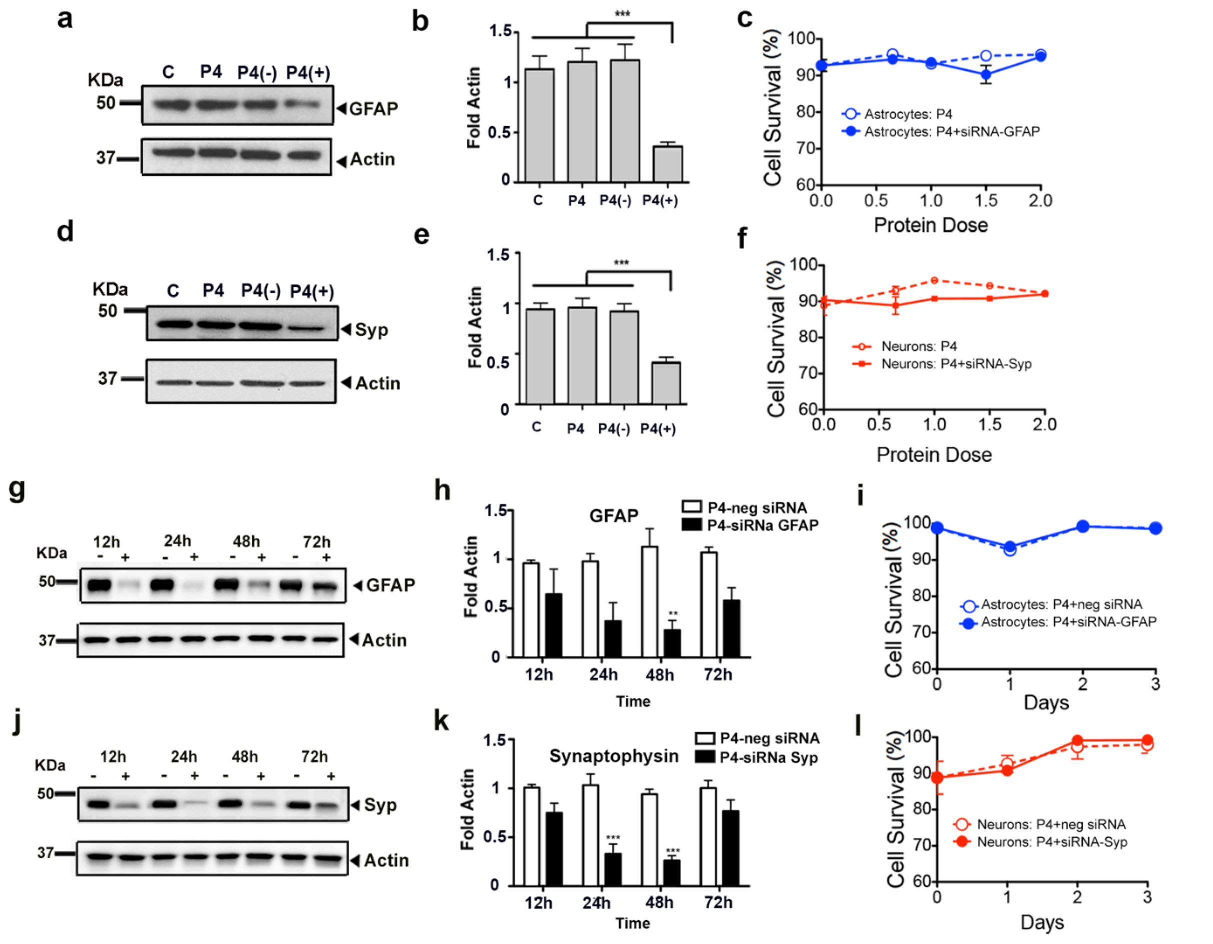

Figure S5. Down-regulation of GFAP and Synaptophysin with supramolecular particles. (a, d) Western blots and (**b, e**) densitometry (intensity values normalized to actin) of **(a, b)** GFAP marker in astroglial cell cultures **(d, e)** and synaptophysin (Syp) in neuronal cultures 24h post-transfection in control condition (C), P4 nanoparticle alone (P4), negative (P4 (-)) and positive P4(+)) siRNA for GFAP or synaptophysin. (A dose of P4-siRNA equivalent to 34 µM- 65 nM was used to transfect cells. **(c,f)** Cell survival of (**c**) astroglial cells and (**f**) neuronal cultures after transfection with four increasing doses of P4 alone (P4) or P4-siRNA (P4 (+))(P4-siRNA: Dose 1; 34 µM-65 nM, Dose 2; 52 µM -100 nM, Dose 3; 78 µM - 150 nM and Dose 4; 104 µM - 200 nM). **(g, j)** Western blots and **(h, k)** densitometry (intensity values normalized to actin) of **(g, h)** GFAP marker in astroglial cells **(j, k)** and synaptophysin in neuronal cultures over the time (12, 24, 48 72 hours).. **(i, l)** Cell survival of **(i)** astroglial cells and **(l)** neuronal cultures after different time points for negative or positive siRNA+P4. Dose 4 was used for the time-course experiments *P <0.05, **P <0.001, LSD test (compared with Control or negative siRNA P4), n= 6 for (a,d) and n=3 (g,j).

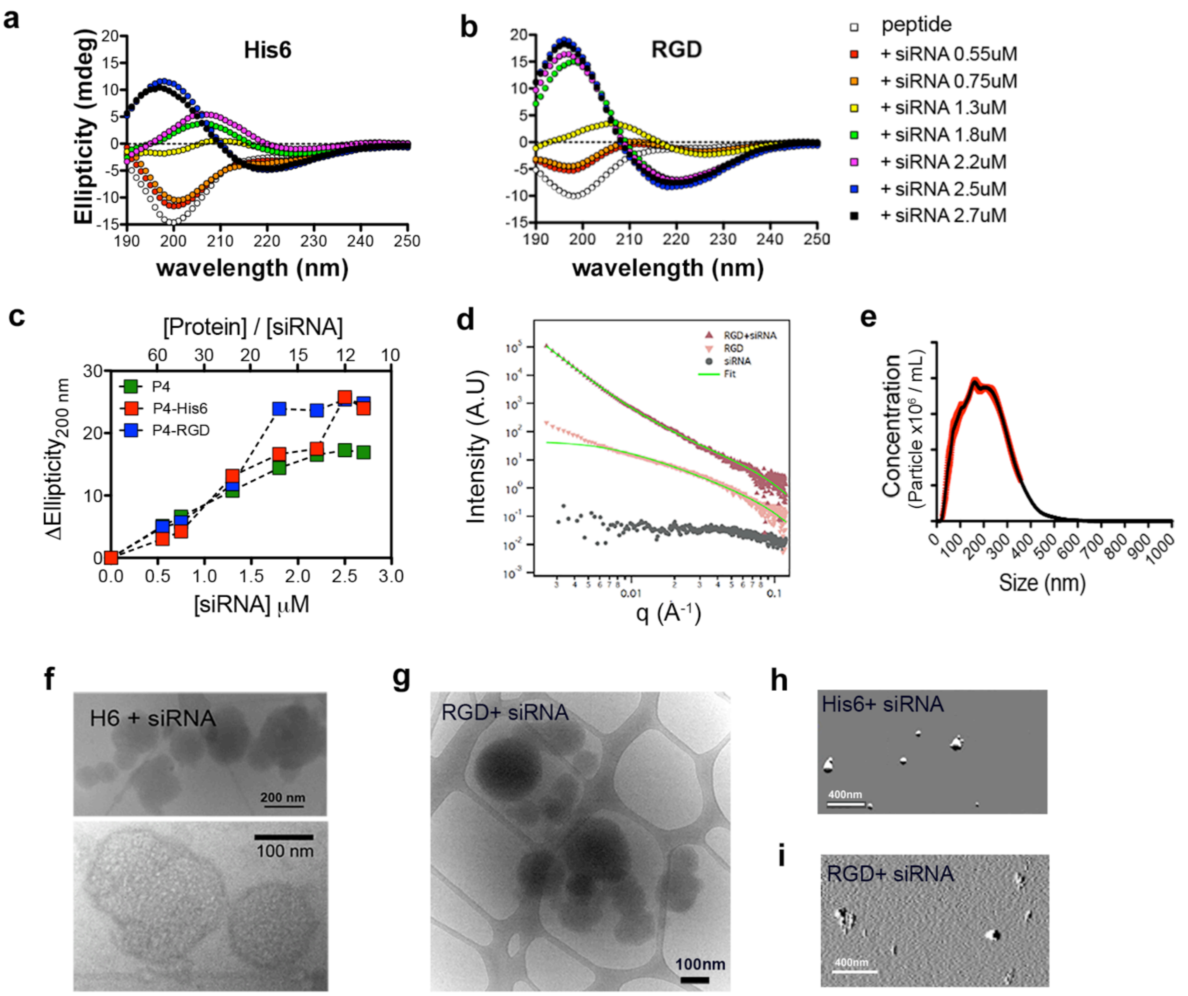

Figure S6. Functionalized peptide-siRNA particles. Circular dichroism of **(a)** P4-His6 and **(b)** P4-RGD with siRNA. **(c)** Ellipticity of P4-His6 and P4-RGD peptides when mixed with siRNA. **(d)** SAXS results of P4-RGD samples and fit of data with a polydisperse sphere model. **(e)** Nanoparticle tracking analysis of P4-RGD + siRNA particles. **(f, g)** Cryo-TEM of **(f)** P4-His6 + siRNA and **(g)** P4-RGD+siRNA particles. **(h, i)** AFM of **(h)** P4-His6 + siRNA and **(i)** P4-RGD+siRNA particles.

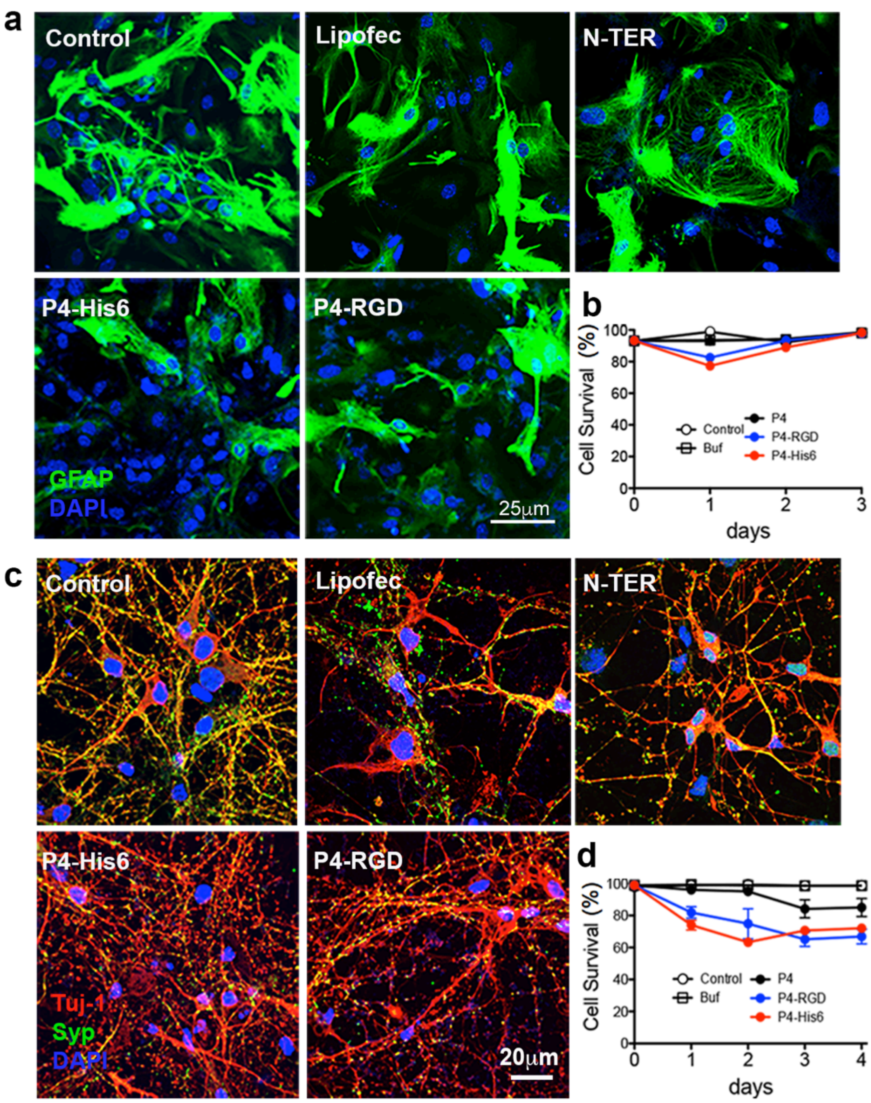

Figure S7. Cell survival with programmable P4 nanoparticles. (a, c) Confocal images of **(a)** glial cells stained with GFAP (green) and DAPI (blue) and **(c)** neuronal cells stained with Synaptophysin (Syp, green), Tuj-1 (red) and DAPI (blue) 24h after transfection. **(b, d)** Cell survival of **(b)** glial cells and **(d)** neuronal cells 4 days post transfection in starvation media (Control), Buffer (Buf), P4 nanoparticle (P4), P4 functionalized with His6 (P4-His6) and P4 functionalized with RGD (P4-RGD).

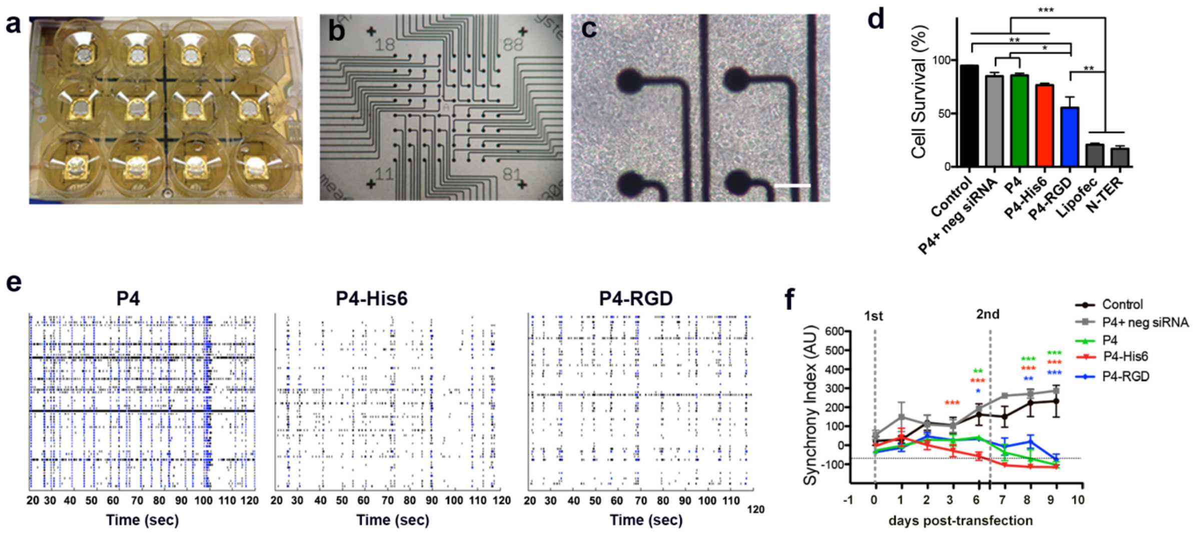

Figure S8. Electrical activity of neural cells transfected with programmable P4 nanoparticles. (a) MEA plate, **(b, c)** bright field of MEA wells (the electrodes are shown in black) and **(c)** neural cells cultured for 15 days. **(d)** Cell survival of neural cells in MEA plates after 2 transfections of siRNA for synaptophysin with P4, P4-His6 or P4-RGD, Lipofectamine and N-TER nanoparticles (siRNA at 200nM). A negative siRNA+P4 nanoparticles and starvation media (Control) were used as controls. **(e)** Raster plots from 64 electrodes of representative P4, P4-His6 and P4-RGD treated MEA wells 3 days post transfection. Each line represents the signals detected by a single electrode of the MEA array, during 100 seconds recordings. **(f)** Synchrony index of neural cells in MEA plates after 2 transfections of Synaptophysin siRNA. Scale Bar: 100 µm. All the experiments were performed using a dose 4 (P4: 104 µM – siRNA: 200 nM). *P <0.05, **P <0.001, LSD test n= 6 wells with 64 electrodes per well per condition were analyzed.

Table S3. Comparison of protein silencing efficacy between present particles and similar systems reported in literature.

Peptide Delivery system	Cell type	Gene/Protein Silenced	% of Silencing	Toxicity	Reference
**P4 Nanoparticles**	Primary cortical astrocytes and neuronal cells	GFAP & Synaptophysin	80-83%	5-15%	Present study
**Rabies virus glycoprotein (RVG) peptide**	Neuro 2a cell line	GFP & Dylight-647	70-75%	<10%	[5, 6]
**Penetratin 1 peptide vector**	Primary hippocampal neurons	SOD-1	>80%	<5%	[7]
**Cyclodextrins (CDs)**	Immortalized and Primary neuronal cells	Luciferase & GAPDH)	40% & 68%	20%	[8]
**Peptide nanofiber (PNFs)**	Primary neuronal cells	BCL2	Not specified	Low (LDH assay)	[9]
**RVG-9dR (Rabies virus glycoprotein peptide fused to 9dR residues)**	Brain microglial cell line (N9)	TNF-α	~60% (derived from graphs)	Not reported	[10]
**homing peptides MG1-9R**	microglia	interferon regulatory factor 5 (IRF5)	60%	Not reported	[11]

REFERENCES

[1] Z. Álvarez, M. A. Mateos-Timoneda, P. Hyroššová, O. Castaño, J. A. Planell, J. C. Perales, E. Engel, S. Alcántara. *Biomaterials* **2013**, *34*, 2221.

[2] M. Mattotti, Z. Alvarez, J. A. Ortega, J. A. Planell, E. Engel, S. Alcántara. *Biomaterials* **2012**, *33*, 1759.

[3] Z. Álvarez, P. Hyroššová, J. C. Perales, S. Alcántara. *Cereb. Cortex* **2016**, *26*, 1046.

[4] J. Adler, *I. Parmryd. Cytom. Part A* **2010**, *77*, 733.

[5] P. Kumar, H. Wu, J. L. McBride, K. E. Jung, M. H. Kim, B. L. Davidson, S. K. Lee, P. Shankar, N. Manjunath. *Nature* **2007**, *448*, 39.

[6] E. J. Kwon, M. Skalak, R. L. Bu, S. N. Bhatia. *ACS Nano* **2016**, *10*, 7926.

[7] T. J. Davidson, S. Harel, V. A. Arboleda, G. F. Prunell, M. L. Shelanski, L. A. Greene, C. M. Troy. *J. Neurosci.* **2004**, *24*, 10040.

[8] A. M. O’Mahony. B. M. D. C. Godinho, J. Ogier, M. Devocelle, R. Darcy, J. F. Cryan, C. M. O’Driscoll. *ACS Chem. Neurosci.* **2012**, *3*, 744.

[9] M. Mazza, M. Hadjidemetriou, I. de Lázaro, C. Bussy, K. Kostarelos. *ACS Nano* **2015**, *9*, 1137.

[10] S.S. Kim, C. Ye, P. Kumar, I. Chiu, S. Subramanya, H. Wu, P. Shankar, N. Manjunath. *Mol. Ther.* **2010**, *18*, 993.

[11] T. Terashima, N. Ogawa, Y. Nakae, T. Sato, M. Katagi, J. Okano, H. Maegawa, H. Kojima. *Mol. Ther. Nucleic Acids* **2018**, *11*, 203.
